# Maternal and partner’s level of satisfaction on the delivery room service in University of Gondar Referral Hospital, northwest, Ethiopia: a comparative cross-sectional study

**DOI:** 10.1186/s12913-020-05079-8

**Published:** 2020-03-19

**Authors:** Asefa Adimasu Taddese, Kiros Terefe Gashaye, Henok Dagne, Zewudu Andualem

**Affiliations:** 1 University of Gondar College of medicine and health science institute of public health department of epidemiology and Biostatistics, Gondar, Ethiopia; 2 University of Gondar College of medicine and health science school of medicine department of gynecology and obstetrics, Gondar, Ethiopia; 3 University of Gondar College of medicine and health science institute of public health department of Environmental and occupational health and safety, Gondar, Ethiopia

**Keywords:** Birth satisfaction scale, Maternal satisfaction, partner’s satisfaction

## Abstract

**Background:**

Asking patients/clients what they perceive about the care and treatment they have received is one of the important steps towards improving the quality of health care. In the scientific world, a number of efforts have been tried to understand about what laboring mothers perceive about the care provided. However, little is known about the birth experiences of partners in Ethiopia. Therefore, this study was aimed to assess the maternal and partner’s level of satisfaction on the delivery room service in the study area.

**Methods:**

A comparative cross-sectional study was conducted from December 2018 to January 2019 in University of Gondar referral hospital. The birth satisfaction scale is used for the mother, and it was adapted to the partners’ perspective. Paired-samples t tests were used for comparing mothers and partners for the birth satisfaction scales global and thematic scores. A binary logistic regression model was fitted to identify predicting factors for mothers’ and partners satisfaction.

**Results:**

The overall satisfaction of mothers in this study was 47.6%. Whereas, 41.2% of partners were satisfied by delivery room services. There were mean difference between mothers and partners’ birth satisfaction scale (*p* = 0.02). Maternal satisfaction scale was affected by age [OR = 0.36, 95%CI (0.18, 0.73)], perception [OR = 0.02, 95%CI (0.001,0.09)], waiting time [OR = 0.11, 95%CI (0.001, 0.09)],visiting mode [OR = 0.01, 95%CI (0.001,0.08)], pregnancy status [OR = 0.04, 95%CI (0.01,0.33)] and fatal outcome [OR = 0.001, 95%CI (0.001,0.018)] .whereas, partners satisfaction was associated with age [OR = 0.16,95%CI (0.05 0.49)], occupational status [OR = 0.02, 95%CI (0.001, 0.24), amount of money to pay for service [OR = 2.87, 95%CI (1.07, 7.71), visiting mode of his wife [OR = 0.08, 95%CI (0.01, 0.35)], waiting time [OR = 0.12, 95%CI (0.04, 0.33)], privacy [OR = 10.61, 95%CI (3.00, 37.52)], mode of delivery of his wife [OR = 7.69, 95%CI (3.00, 19.69)].

**Conclusion:**

This finding would alert the health care system to design a client-friendly approach. It will provide insight to hospital administrators and providers in formulating a policy that would enhance the support of partners during labour and delivery process.

## Background

Since in the early 1990s the health care executives and suppliers have started to evaluate patient’s satisfaction; complaints and concerns about the provision of care have started to rise. That is why the patient satisfaction surveys have become an integral part of hospital and clinical management strategies in today’s world [1, 2].

Asking patients what they perceive about the care and treatments provided is a significant step towards improvement of the quality of care if it is culminated towards solving the identified patient complaints [3]. Doing such activities can be one of the fundamental milestones in delivering qualified health care by health institutions and in ensuring whether community demands are met. It is a reality that satisfaction affects the fact that an individual is seeking medical advice and has an ongoing connection with a practitioner [4].

In the past, the male person was associated with his work outside of the home and the mother’s work in the house and caring for children. This traditional way of job assignment have led to fathers and mothers traditionally have distinct functions in some part of the world even today [3].

Currently, especially in the developed world, both field and home activities are becoming shared responsibilities of all family members particularly the mother and the father [5]. In developing countries like Ethiopia, sharing of all form of responsibilities is increasing and fathers are increasingly coming to attend the labor of their wives [6].

The satisfaction of the mother through the birth cycle is the measure of quality assessment of maternity services most frequently mentioned [7, 8]. A favorable birth experience is linked to the enhanced mother-to-child and maternal skills and adds to its sense of achievement and self-esteem [9–12]. In contrast, a negative experience in birth can make the mother feel distressed and has adverse effect on her mental health, increasing the risk of postpartum depression and post-traumatic stress disorder [13–15]. The primary reasons why the health system is being used less often are negative experiences that can lead to an enhanced birth cycle associated with complications, including death of mothers [13–15]. In the science literature, parents have their own favorable emotions of birth: fatherhood or child pride and love and thankfulness to their partner [16]. In a male dominant community like Ethiopia, the role of fathers on the labor and delivery process including where to labor and delivery of their wives is quite significant [16]. In such condition dissatisfied fathers can negatively influence future utilization of the health system with a potential risk to the mother’s future life [16, 17]. Although fathers ‘birth participation in Ethiopia has been lately increasing, their emotions and experiences were not widely researched. Few studies have been conducted that focus on satisfaction of the women and their partners simultaneously [18, 19]. Knowing how satisfied partners are and how would determine their future births can provide insight for health professionals and managers as to how parental care and services can be evaluated and adapted during the birth process. This research was therefore designed to assess and compare the level of satisfaction of mothers and partners and to identify the determinant factors of satisfaction during the birth process.

## Methods

### Study design, area and period

A comparative cross-sectional study design was conducted from December 2018 to January 2019 in University of Gondar referral hospital. The hospital is found about 750 km northwest of Addis Ababa (the economic capital of Ethiopia). It has more than 500-bed capacity. The hospital is used as the referral center for more than 7 million catchment population. It provides both specialty and subspecialty services ‘including pediatrics, surgery, gynecology and obstetrics, internal medicine, psychiatry, ophthalmology, etc. in its inpatient and outpatient clinics. According to hospital’s data registry, more than 10,000 mothers delivery service annually [20].

### Source and study population

All laboring mothers and their partners who visited in University of Gondar referral Hospital for the delivery services were the source population. All mothers with their partners who gave birth and served in the referral Hospital and full fill the selection criteria during data collection period were the study population.

#### Inclusion criteria

Mothers with their partners who attended delivery services in the study hospital.

#### Exclusion criteria

Mothers and/or their partners who were mentally or critically ill and unable to communicate.

### Sample size determination and sampling technique

Simple random sampling techniques were used to select the sample. Mothers and Partners were recruited postpartum, at least 6 h after vaginal birth and 12 h after cesarean delivery considering they have got better rest and stability and the required sample size was determined by the comparative cross-sectional sample size formula by considering the following assumptions. Expected proportion (*p* = 0.62) for maternal satisfaction rate from the previous study [21]. However, no previous study was conducted about paternal satisfaction; therefore, we used 50% proportion with a 95% confidence level and 80% power.

Therefore, the required sample size (n) for this study was calculated by using comparing two Independent Proportions (p) formula as follows;

$$ n=\frac{p_1\left(1-{p}_1\right)+{p}_2\left(1-{p}_2\right)}{{\left({p}_1-{p}_2\right)}^2}\times f\left(\upalpha, \upbeta \right) $$

Based on the assumption, the calculated sample size (no) was

$$ n=\frac{0.62\left(1-0.62\right)+0.5\left(1-0.5\right)}{{\left(0.62-0.5\right)}^2}\times 7.85=265 $$

And, adding 11% for non-response rate during the actual study, then the sample size become 294.

### Data collection and analysis

The self-administered questionnaire (Supplementary file 1), which included socio-demographic variables and the birth satisfaction scale (BSS) and the birth medical report was examined. The BSS is a validated 30-point survey designed to assess satisfaction in 15 different areas [22, 23]. Using this instrument, satisfaction was measured using a series of simple statements with five-level Likert scales that requests participants to rate their level of agreement with each item (0 = Strongly Disagree, 1 = Disagree, 2 = Neither Agree or Disagree; 3 = Agree; 4 = Strongly Agree) (almost half of the items are reverse-scored), which results in a total score of 120 [24].

The five level Satisfaction tool responses were translated to satisfied and dissatisfied. Consequently, the very satisfied and satisfied responses were combined as satisfied and the very dissatisfied, dissatisfied and neutral responses were turned into dissatisfied. Neutral answers have been graded as dissatisfied by considering the respondents might be a fearful way to express themselves. The total satisfaction level was classified based on the average/mean value of all items; people who answered questions above the mean value were taken as satisfied and below the mean value were dissatisfied.

BSS measurements were adapted to partner’s circumstances and BSS questionnaires that were less than two-thirds completed were rejected and others were scaled to obtain a total score of 120.

We used Stata 14 software for all statistical analysis. The mean and SD for continuous variables were calculated and a proportion for categorical variables is indicated. For a mean comparison, t-tests in pairs for the global and thematic BSS scores were conducted. The logistic regression analysis was adapted in order to identify important predictors and variables with *p*-value less than 0.05 was interpreted as significant factors. Most of the categorical variables have two category and the last categories were selected as a reference group and interpreted as like a unit difference because of within the category there is no that much significant difference and in logistic regression analysis when significant difference was not observed between the category, no need of selecting reference [25].

## Results

### Socio-demographic characteristics

A total of 294 participants were targeted for this study. Of these, 294 participants were enrolled with a response rate of 100%. Their mean age of mothers and their partners’ were 27.4 and 32.8 respectively, 83.67% of mothers and partners were educated formally. The majority (55.1%) of mothers were unemployed while only 31(10.54%) partners’ were unemployed. Two hundred and 42 (82.3%) of mother’s perceive the presence of waiting area around the delivery room and 58.1% of mothers were waited more than 1 h before seeing doctors (Table 1).

**Table 1:** Socio-demographic characteristics of study participants’ at University of Gondar referral hospital from Dec 2018 to Jan. 2019 (*n* = 294)

Characteristics		Mothers frequency (n = 294)		Partners frequency (n = 294)
Age	18–20	13 (4.42)	18–20	4 (1.36)
	20–24	103 (35.03)	20–30	137 (46.60)
	25–29	75 (25.51)	30–40	151 (51.36)
	30–35	51 (17.35)	Greater than 40	2 (0.68)
	Greater than 35	52 (17.69)		
Educational status	Educated	246 (83.67)	246 (83.67)	
	Uneducated	48 (16.33)	48 (16.33)	
Occupational status	Employed	132 (44.90)	263 (89.46)	
	Unemployed	162 (55.10)	31 (10.54)	
Religion	Orthodox	239 (81.29)	231 (78.57)	
	Muslim	21 (7.14)	29 (9.86)	
	Protestant	34 (11.56)	34 (11.56)	
Marital status	Married	277 (94.22)	266 (90.48)	
	Single	9 (3.06)	20 (6.80)	
	Divorced	8 (2.72)	8 (2.72)	
Average amount of money paid for service (ETB)	less than 157	224 (76.19)		
	greater than 157	70 (23.81)		
Mother’s perception due to the presence of waiting area	Yes	242 (82.31)		
	No	52 (17.69)		
Waiting time before seeing doctors (Hr)	> 1 h	171 (58.16)		
	< 1 h	123 (41.84)		
Privacy during the medical examination	Yes	264 (89.80)		
	No	30 (10.20)		

### Obstetrical characteristics of mothers

As the delivery character of women (Table 2), 52.7% of parturient visited the hospital after referred from other health institutions. Majorities of parturient (71.1%) delivered in the hospital for the first time. Two hundred and 54 (86.4%) of pregnancy was wanted and only 13.6% pregnancy unwanted. Among born babies, 94.9% were live birth and cesarean delivery covered the majority (53.7%) mode of delivery.

**Table 2:** Obstetrical characteristics of mothers at University of Gondar referral hospital from Dec. 2018 to Jan. 2019 (n = 294)

Delivery character		Frequency (%)
Mode of visiting	Referral	155 (52.7)
	Not referral	139 (47.3)
Parity	Primi-para	209 (71.1)
	Multipara	85 (28.9)
Wanted status of pregnancy	Wanted pregnancy	254 (86.4)
	Unwanted pregnancy	40 (13.6)
Mode of delivery	Spontaneous vaginal delivery	49 (16.7)
	Assisted delivery	87 (29.6)
	Cesarean section	158 (53.7)
Fatal outcome	Live birth	279 (94.9)
	Stillbirth	15 (5.1)

### Mean comparison of mothers and partners level of satisfaction

When we analyzed the mean differences between the mothers’ and partners’ satisfaction level, we observed that the mothers considered themselves more prepared (*p* < 0.001) and more supported by families (p < 0.001). Meanwhile, there was no mean difference between mothers and partners satisfaction with distress experienced during labor/labor (*p* = 0.12) and receipt of an obstetric intervention (*p* = 0.89), as a whole there was the mean difference between mothers and partners’ birth satisfaction scale (*p* = 0.02) (Table 3).

**Table 3:** Paired t-test mean comparison of participants’ at University of Gondar referral hospital from Dec. 2018 to Jan. 2019 (n = 294)

Themes of birth satisfaction score	Subthemes of birth satisfaction score	Mean satisfaction scores (SD)		Mean difference	p-Value
		Mothers	Partners		
Quality of Care provision (QC)	Home assessment	0.517 (0.500)	0.629 (0.483)	−0.112	0.004
	Birth environment	0.146 (0.353)	0.251 (0.434)	−0.105	0.001
	Sufficient support	0.108 (0.311)	0.251 (0.434)	−0.142	0.0001
	Relationships with health care professionals	0.278 (0.449)	0.166 (0.373)	0.112	0.0001
Women’s Attributes (WA)	Ability to cope during labour	0.782 (0.413)	0.234 (0.424)	0.547	0.0001
	Feeling in control	0.323 (0.468)	0.829 (0.376)	−0.506	0.0001
	Preparation for childbirth	0.755 (0.430)	0.663 (0.473)	0.092	0.010
	Relationship with baby	0.081 (0.274)	0.261 (0.440)	−0.180	0.0001
Stress experienced	Distress experienced during labour	0.378 (0.485)	0.320 (0.467)	0.058	0.129
	Obstetric injuries	0.537 (0.499)	0.731 (0.444)	−0.193	0.0001
	Perception of having received sufficient medical care	0.530 (0.499)	0.799 (0.401)	−0.268	0.0001
	Receipt of an obstetric intervention	0.833 (0.373)	0.836 (0.370)	−0.003	0.895
	Pain experienced	0.972 (0.162)	0.748 (0.434)	0.224	0.0001
	Long labour	0.758 (0.428)	0.894 (0.571)	−0.136	0.0001
	Health of baby	0.537 (0.499)	0.3571 (0.479)	0.180	0.0001
Total satisfaction		0.524 (0.500)	0.588 (0.492)	−0.064	0.020

Only 35.4% of mothers and 42.9% of their partners were satisfied by the overall quality of care provision, satisfaction with women’s personal attribute were higher in partners’ (55.1%) compared with mothers (42.9%) and 34.0% mothers and 33.7% partners were satisfied by stress experienced during labour/labor. Generally, 47.6% of the mothers and 41.2% of their partners were satisfied with the overall service of the delivery room in Gondar university referral hospital (Table 4).

**Table 4:** maternal and partners level of satisfaction at University of Gondar referral hospital from Dec 2018 and Jan 2019 (n = 294)

Birth satisfaction scale themes	Maternal Level of satisfaction, Frequency (%)		Partners Level of satisfaction, Frequency (%)	
	Satisfied	dissatisfied	Satisfied	Dissatisfied
Quality of Care provision (QC)	104 (35.4)	190 (64.6)	126 (42.9)	168 (57.1)
Women’s Attributes (WA)	126 (42.9)	168 (57.1)	162 (55.1)	132 (44.9)
Stress experienced during Labour (SL)	100 (34.0)	194 (66.0)	99 (33.7)	195 (66.3)
Overall satisfaction	140 (47.6)	154 (52.4)	121 (41.2)	173 (58.8)

### Factors associated with satisfaction of mothers in delivery room services

Maternal satisfaction with delivery care was associated with age [OR = 0.36, 95%CI (0.18, 0.72)], religion [OR = 3.14, 95%CI (1.51, 6.51)], the perception about the presence of waiting area [OR = 0.01, 95%CI (0.001,0.09)], waiting time before seeing a doctor or a nurse [OR = 0.11,95%CI (0.001, 0.09)],visiting mode [OR = 0.01, 95%CI (0.001,0.08)], pregnancy status [OR = 0.04, 95%CI (0.001,0.33)], fatal outcome [OR = 0.001, 95%CI (0.001,0.07)] were statistically significant effects.

Keeping all other variables constant; the odds of the maternal level of satisfaction were increased by 0.36 times [OR: 0.36, 95% CI (0.18, 0.72)]; for a unit difference of the age of mothers. Mothers who waited more than 1 h before doctors seen were 0.11 times more satisfied than waited less than 1 h [OR: 0.11, 95% CI (0.001,0.09)] (Table 5).

**Table 5:** Factors associated with satisfaction of mothers at University of Gondar referral hospital from Dec 2018 and Jan 2019 (n = 294)

Variable’s	OR	SE.	Z	P > z	[95% Conf. Interval]	
					Lower	Upper
Age of Mothers’	0.361	0.128	−2.86	0.004^**^	0.180	0.725
Educational status of Mothers	11.75	15.79	1.83	0.067	0.843	163.78
Occupation status of Mothers	47.14	31.43	5.78	0.000^***^	12.761	174.20
Religion of Mother	3.143	1.168	3.08	0.002^**^	1.517	6.511
Average monthly income (ETB)	0.369	.3913	−0.94	0.347	0.046	2.946
Amount paid to the service (ETB)	2.756	1.731	1.61	0.106	0.804	9.438
Perceived presence of waiting area	0.017	.0147	−4.82	0.0001^***^	0.003	0.091
Visiting mode	0.012	.0122	−4.57	0.000^***^	0.001	0.083
Waiting time before seeing a doctor or a nurse	0.110	.0707	−3.45	0.001^***^	0.032	0.387
Privacy during examinations	67.49	120.0	2.37	0.018^**^	2.068	2202.4
Number of parity	162.2	165.0	5.00	0.0001^***^	22.112	1190.8
Pregnancy status	0.044	0.045	−3.04	0.002^**^	0.006	0.330
Mode of delivery	2.458	1.228	1.80	0.072	0.923	6.547
Immediate maternal condition after delivery	0.579	0.439	−0.72	0.472	0.131	2.564
Fatal outcome	0.001	0.003	−3.31	0.001^***^	0.000	0.074

*** = **variables significant at the 0.01 level**

** = **variables significant at the 0.05 level**

### Factors associated with satisfaction of partners in delivery room services

Partners’ satisfaction with delivery care was associated with age [OR = 0.161, 95%CI (0.053 0.496)],religion [OR = 3.584, 95%CI (1.783,7.205), occupational status [OR = 0.027, 95%CI (0.003, 0.239), average monthly income [OR = 0.990, 95%CI (0.990, 0.999), amount of money to pay for service [OR = 2.87, 95%CI (1.068, 7.713),visiting mode of his wife/client [OR = 0.082, 95%CI (0.019, 0.346), waiting time of his wife/clients before seeing a doctor or a nurse [OR = 0.123, 95%CI (0.044, 0.334), care providers measure taken to assure privacy during examinations [OR = 10.611, 95%CI (3.001, 37.524), mode of delivery of his wife/client [OR = 7.689, 95%CI (3.002, 19.696) were statistically significant factors.

Keeping all other variables constant; the odds of parents satisfaction were increased by 0.161 times [OR: 0.161, 95% CI (0.053, 0.496)]; for one unit increments of the age of families. Partners who follow orthodox Christian were 3.584 times more satisfied than other religion followers [OR: 3.584, 95% CI (1.783, 7.205)] (Table 6).

**Table 6:** Factors associated with satisfaction of partners at UOGRH from Dec 2018 and Jan 2019 (N = 294)

Variables	Odds Ratio	Std. Err.	Z	P > z	[95% CI]	
					Lower	Upper
Age of partners	0.161	0.092	−3.19	0.001^***^	0.053	0.496
Marital status of partners	0.540	0.321	−1.04	0.300	0.168	1.730
Religion of partners	3.584	1.277	3.58	0.000^***^	1.783	7.205
Occupational status of partners	0.027	0.030	−3.25	0.001^***^	0.003	0.239
Average monthly income (ETB)	0.995	0.000	−2.56	0.010^**^	0.994	0.999
Service paid (ETB)	2.87	1.447	2.09	0.037^**^	1.068	7.714
Visiting mode of his wife/attendant	0.082	0.060	−3.41	0.001^***^	0.019	0.346
Waiting time before seeing a doctor or a nurse his wife/attendant	0.121	0.062	−4.09	0.000^***^	0.044	0.333
privacy during examinations	10.611	6.838	3.67	0.000^***^	3.001	37.524
Number of parity	83.745	84.306	4.40	0.000^***^	11.642	602.369
Mode of delivery of his wife	7.6895	3.690	4.25	0.000^***^	3.001	19.696
maternal condition after delivery	.97789	.5232	−0.04	0.967	.34262	2.7910
Fatal outcome	.00324	.01213	−1.53	0.125	2.13e-06	4.9480

*** = **variables significant at the 0.01 level**

** = **variables significant at the 0.05 level**

## Discussion

The study was aimed to evaluate and compare the level of satisfaction of mothers and partners on their birth experience. It also has the aim of correlating their level of satisfaction with the socio-demographic and delivery character of participants.

The overall maternal and partner’s level of satisfaction with the labour and delivery service University of Gondar referral hospital was 47.6 and 41.2%, respectively. In this study, mothers were more satisfied than partners in the overall service of the hospital. This result is in line with other studies [24–26]. According to some evidences, level of satisfaction is affected by level of education and economic status where the higher the economic level and educational status, the lower is the level of satisfaction and vice-versa which may be the reason to explain this finding since Ethiopia is one of the country with poor economic status and low level of education. The second reason might be during childbirth, mothers are directly involved in labor and delivery process which is mainly stressful and leads the mother to the lack of concentration on other factors which can affect her level of satisfaction. In contrast, partners are more conscious and witness of childbirth process and hence they are more likely to be unsatisfied with what they have observed. In addition most of the time, partners were not allowed to enter into the delivery room to support their clients which might have contributed to their low level of satisfaction.

The level of maternal satisfaction in this study was lower than studies conducted in Assela teaching and referral hospital (80.7%) [27], Jimma, Ethiopia (77.0%) [28], Irbid, North Jordan (64%) [29], Côte d’Ivoire (92.5%) [30],Nairobi, Kenya (56%) [31], Sri Lanka (48%) [32],Mekelle, Ethiopia (79.7%) [33], Black Lion Referral Hospital Addis Ababa, Ethiopia (90.1%) [34]. However, higher than studies conducted in Asmara Public Hospitals, Eritrea 20.8% [35]. This variation may be because of a real difference in the quality of services provided or difference in the socio-demographic and study setting of countries [36, 37]. The lowest proportion in the study area may due to in the current situation the hospital have had greater patient flow beyond capacity (more than 7 million people catchment population as compared to 3.5 million people according to Health Sector Development Plan -IV plan) [38], which could impede the general qualities of the health care deliveries, and mothers admitted to obstetrics and gynecology wards.

Older mothers (*p* = 0.004) were 0.36 times more satisfied than the new mothers. This is supported by other study [27]. This might be related to as the age of mother increase, the stability of life situation increase and more prepared for medical and psychological stresses. In addition, more aged mothers have many exposures for childbirth and comparing the current delivery from the past they might be more satisfied because maternal and child health status was modified from the past.

In this research both mothers and associates who adopt the Islam and Protestant religions were more satisfied than orthodox Christians. The reason could be more co-ordinates and endorsed support by their families within traditional or spiritual views of their respective religions [39].

Mothers who have had work were 47.14 times more satisfied than unemployed mothers. Such finding may be due to the fact that, mothers having job feel economically secured and happier to enjoy child birth than their counterparts.

It has been noted that mothers who wished pregnancy were more likely to be satisfied than those mothers who had an unwanted pregnancy. A similar finding was reported in Arsi, Ethiopia and Nairobi, Kenya [27, 31].

Mothers who reported privacy during the physical examination were more satisfied than those who perceived the absence of privacy. Other researches also indicated that mothers who admitted for reproductive organ related issue that is a sensitive organ, that also contributes a sense of shame to the physical examination process, thereby increasing the discomfort of women and reducing their levels of satisfaction if privacy is not retained [28, 33, 40, 41].

The partner level of satisfaction was affected by the amount of money paid for service, fathers who paid less than or equal to157 ETB were more satisfied than those who paid greater than157 ETB. The result was supported by studies conducted in Amhara regional state [40]. This might be due to the low socioeconomic status of families. Labor and delivery care is freely served in the Hospital. But families may be requested to by some medications and supplies in case of shortage. Such unexpected costs might negatively influence the level of satisfaction. However, mothers’ level of satisfaction was not affected by the amount of money paid for service, it might be related with the majority of mothers (55.10%) were unemployed and partners are responsible for the expenditure.

Mothers who have had one-childbirth (71.1%) were more satisfied than those who had more than two (28.9%) childbirths. This finding is in line with other studies [27, 33, 42–44]. But in contrast to the study conducted in Iraq where mothers who had more than two births were more satisfied than those who had one childbirth [45]. This might be due to the fact that, new mothers are egger to have the first child hence felt satisfied than the previous women. The variation in the previous study outcomes may be due to the difference in the country’s’ economic status and service quality.

## Conclusion

The finding of this research indicated that the overall level of satisfaction of mothers and their partners is very low compared to other similar hospitals in the country. In addition, mothers were more satisfied than partners’ by delivery room service. Age, religion, perception, waiting time, visiting mode, parity, pregnancy status, and fetal outcome were important predictors of maternal and partners level of satisfaction in delivery room services. So, the findings of our study would alert the health care system to design a partner-friendly approach to enhance the very low level of satisfaction of the Hospital both to the mother and her partner.

Even though, recently the majority of mothers were delivered in the health institutions clients in our country complain about the services. Evaluate health care services from the patient’s point of view, giving clients centered services and targeting to identify problem areas are important. Therefore researchers should invest their time in these areas of the country by conducting research for the future.

## Supplementary information

**Additional file 1.** Maternal and partenars level of satisfaction in the delivery room Gondar university referral hospital Northwest Ethiopia, 2019.

## Data Availability

The data upon which the result based could be accessed a reasonable request to the corresponding author.

## Abbreviations

BSS: Birth Satisfaction Scale
CI: Confidence Interval
OR: Odds Ratio
SD: Standard Deviation
UOGRH: University of Gondar Referral Hospital
