# Supplementary material for: Maternal and partner’s level of satisfaction on the delivery room service in University of Gondar Referral Hospital, northwest, Ethiopia: a comparative cross-sectional study
Source: BMC Health Serv Res. 2020 Mar 19;20:233. doi: 10.1186/s12913-020-05079-8 (PMC7083028; doi:10.1186/s12913-020-05079-8)
Supplement: Supplementary file 1 — Additional file 1. Maternal and partenars level of satisfaction in the delivery room Gondar university referral hospital Northwest Ethiopia, 2019. [file 12913_2020_5079_MOESM1_ESM.docx]

Maternal and partenars level of satisfaction in the delivery room Gondar university referral hospital Northwest Ethiopia, 2019:

**Part I: Socio-demographic questions for mother**

| 1. **Age_________** | 1. **Educational status** 2. Illiterate 3. B.litrate | 1. **Occupational status** 2. Unemployed 3. B. Employed |
| --- | --- | --- |
| 1. **Religion**    1. Orthodox    2. Muslim    3. Protestant | 1. **Marital status** 2. Married 3. Single 4. Divorce | 1. **Amount paid to the servic________** |
| 1. **Average household monthly income____________** | 1. **Residence** 2. Urban 3. Rural | 1. **Perceived presence of waiting area** 2. Yes 3. .No |
| 1. **Mode of visit** 2. Referral 3. Not referral | 1. **Waiting time before seeing a doctor or a nurse** 2. ≤ 1 hour 3. > 1 hours | 1. **Privacy during examinations** 2. Yes 3. No |

**Part II: Delivery characteristics of mother**

| 1. Parity (number including the new baby)   ___________ | 1. Reason for visit 2. Planned delivery 3. Referral delivery | 1. Mode of delivery 2. Spontaneous vaginal delivery (SVD) 3. Assisted delivery 4. Caesarean section(C/S) |
| --- | --- | --- |
| 1. Wanted status of pregnancy 2. Wanted 3. Unwanted | 1. Immediate maternal condition after delivery 2. Normal 3. With complications | 1. Fetal outcome 2. Live birth 3. Stillbirth |

**Part III: Maternal satisfaction on delivery service**

|  |  | **Likert scales** | | | | |
| --- | --- | --- | --- | --- | --- | --- |
|  |  | VS | S | N | D | VD |
|  | **Quality of Care Provision** |  |  |  |  |  |
|  | I felt well supported by my partner during labor and birth. |  |  |  |  |  |
|  | My birth experience was considerably different to what I intended. |  |  |  |  |  |
|  | I had the same midwife/nurse throughout the entire process of labor and delivery. |  |  |  |  |  |
|  | I felt that the delivery room was unthreatening and comfortable. |  |  |  |  |  |
|  | I felt well supported by staff during my labor and birth. |  |  |  |  |  |
|  | My birth proceeded as I planned it |  |  |  |  |  |
|  | The staff communicated well with me during labor. |  |  |  |  |  |
|  | The delivery room was clean and hygienic. |  |  |  |  |  |
|  | 1. **Women’s Personal Attribute** |  |  |  |  |  |
|  | I coped well during my birth. |  |  |  |  |  |
|  | The delivery room staff encouraged me to make decisions about how I wanted my birth to progress. |  |  |  |  |  |
|  | I was well prepared for my labor (i.e., read a lot of literature and/or attended parenthood education classes). |  |  |  |  |  |
|  | I felt very anxious during my labor and birth. |  |  |  |  |  |
|  | I felt out of control during my birth experience. |  |  |  |  |  |
|  | I felt it was better not to know in advance about the processes of giving birth. |  |  |  |  |  |
|  | I was encouraged to hold my baby for a substantial amount of time after birth. |  |  |  |  |  |
|  | I was separated from my baby for a considerable period of time after my birth. |  |  |  |  |  |
|  | **Stress Experienced During Labor** |  |  |  |  |  |
|  | I found giving birth is a distressing experience. |  |  |  |  |  |
|  | I came through childbirth virtually unharmed. |  |  |  |  |  |
|  | I gave birth to a healthy normal baby. |  |  |  |  |  |
|  | During labor I received outstanding medical care. |  |  |  |  |  |
|  | I received a lot of medical intervention, (i.e., induction, forceps, section etc). |  |  |  |  |  |
|  | I had a swift and speedy labor. |  |  |  |  |  |
|  | I was not distressed at all during labor. |  |  |  |  |  |
|  | I felt mutilated by my birth experience. |  |  |  |  |  |
|  | My baby was avoidably hurt during birth. |  |  |  |  |  |
|  | The staff provided me with insufficient medical care during my birth. |  |  |  |  |  |
|  | I had a natural labor, i.e., minimal medical intervention. |  |  |  |  |  |
|  | I thought my labor was excessively long. |  |  |  |  |  |
|  | Giving birth was incredibly painful. |  |  |  |  |  |

**Part IV: Socio-demographic questions for partners**

| 1. Age_______________ | 1. Marital status 2. Married 3. Single 4. Divorced | 1. Educational status    1. Illiterate    2. literate |
| --- | --- | --- |
| 1. Religion   A. Orthodox  B. Muslim  C. protestant | 1. Average household monthly income__________ | 1. Occupational status 2. Employed 3. .unmployed |

**Part V: Parteners Satisfaction on Delivery Service**

|  |  | Likert scales | | | | |
| --- | --- | --- | --- | --- | --- | --- |
|  |  | VS | S | N | D | VD |
|  | 1. **Quality of Care Provision** |  |  |  |  |  |
|  | I felt well supported my wife during labor and birth. |  |  |  |  |  |
|  | Her birth experience was considerably different to what I intended |  |  |  |  |  |
|  | She had the same midwife/nurse throughout the entire process of labor and delivery |  |  |  |  |  |
|  | I felt that the delivery room was unthreatening and comfortable. |  |  |  |  |  |
|  | I felt she was well supported by staff during her labor and birth. |  |  |  |  |  |
|  | Her birth proceeded as we planned it |  |  |  |  |  |
|  | The staff communicated well with her during labor. |  |  |  |  |  |
|  | The delivery room was clean and hygienic. |  |  |  |  |  |
|  | 1. **Women’s Personal Attribute** |  |  |  |  |  |
|  | I coped well during her birth. |  |  |  |  |  |
|  | The delivery room staff encouraged her to make decisions about how she wanted her birth to progress. |  |  |  |  |  |
|  | I was well prepared for her labor (i.e.(1)read a lot of literature and/or attended parenthood education classes). |  |  |  |  |  |
|  | I felt very anxious during her labor and birth. |  |  |  |  |  |
|  | I felt out of control during her birth experience. |  |  |  |  |  |
|  | I felt it was better not to know in advance about the processes of giving birth. |  |  |  |  |  |
|  | She was encouraged to hold her baby for a substantial amount of time after birth. |  |  |  |  |  |
|  | She was separated from her baby for a considerable period of time after her birth. |  |  |  |  |  |
|  | **Stress Experienced During Labor** |  |  |  |  |  |
|  | I found giving birth is a distressing experience. |  |  |  |  |  |
|  | She came through childbirth virtually unharmed. |  |  |  |  |  |
|  | She gave birth to a healthy normal baby. |  |  |  |  |  |
|  | During labor she received outstanding medical care. |  |  |  |  |  |
|  | She received a lot of medical intervention, (i.e., induction, forceps, section etc). |  |  |  |  |  |
|  | She had an immediate and speedy labor. |  |  |  |  |  |
|  | She was not distressed at all during labor. |  |  |  |  |  |
|  | She felt mutilated by her birth experience. |  |  |  |  |  |
|  | The staff provided her with insufficient medical care during her birth. |  |  |  |  |  |
|  | She had a natural labor, i.e., minimal medical intervention. |  |  |  |  |  |
|  | I thought her labor was excessively long. |  |  |  |  |  |
|  | Giving birth was incredibly painful. |  |  |  |  |  |
|  | Her Labor was not as painful as I imagined |  |  |  |  |  |

VS-very satisfied, S-satisfied, N-neutral, D-dissatisfied, VD-very dissatisfied
